# Clear Genetic Distinctiveness between Human- and Pig-Derived *Trichuris* Based on Analyses of Mitochondrial Datasets

**DOI:** 10.1371/journal.pntd.0001539

**Published:** 2012-02-21

**Authors:** Guo-Hua Liu, Robin B. Gasser, Ang Su, Peter Nejsum, Lifei Peng, Rui-Qing Lin, Ming-Wei Li, Min-Jun Xu, Xing-Quan Zhu

**Affiliations:** 1 State Key Laboratory of Veterinary Etiological Biology, Key Laboratory of Veterinary Parasitology of Gansu Province, Lanzhou Veterinary Research Institute, Chinese Academy of Agricultural Sciences, Lanzhou, Gansu Province, People's Republic of China; 2 College of Veterinary Medicine, Hunan Agricultural University, Changsha, Hunan Province, People's Republic of China; 3 Department of Veterinary Science, The University of Melbourne, Werribee, Victoria, Australia; 4 College of Veterinary Medicine, South China Agricultural University, Guangzhou, Guangdong Province, People's Republic of China; 5 Departments of Veterinary Disease Biology and Basic Animal and Veterinary Science, University of Copenhagen, Frederiksberg, Denmark; 6 Department of Parasitology & Clinical Parasitology, Guangdong Medical College, Zhanjiang, Guangdong Province, People's Republic of China; 7 Department of Veterinary Medicine, Agricultural College, Guangdong Ocean University, Zhanjiang, Guangdong Province, People's Republic of China; 8 College of Animal Science and Veterinary Medicine, Heilongjiang Bayi Agricultural University, Daqing, Heilongjiang Province, People's Republic of China; University of Melbourne, Australia

## Abstract

The whipworm, *Trichuris trichiura*, causes trichuriasis in ∼600 million people worldwide, mainly in developing countries. Whipworms also infect other animal hosts, including pigs (*T. suis*), dogs (*T. vulpis*) and non-human primates, and cause disease in these hosts, which is similar to trichuriasis of humans. Although *Trichuris* species are considered to be host specific, there has been considerable controversy, over the years, as to whether *T. trichiura* and *T. suis* are the same or distinct species. Here, we characterised the entire mitochondrial genomes of human-derived *Trichuris* and pig-derived *Trichuris*, compared them and then tested the hypothesis that the parasites from these two host species are genetically distinct in a phylogenetic analysis of the sequence data. Taken together, the findings support the proposal that *T. trichiura* and *T. suis* are separate species, consistent with previous data for nuclear ribosomal DNA. Using molecular analytical tools, employing genetic markers defined herein, future work should conduct large-scale studies to establish whether *T. trichiura* is found in pigs and *T. suis* in humans in endemic regions.

## Author Summary

Trichuriasis is a neglected tropical disease (NTD) caused by parasitic nematodes of the genus *Trichuris* (Nematoda), causing significant human and animal health problems as well as considerable socio-economic consequences world-wide. Although *Trichuris* species are considered to be relatively host specific, there has been significant controversy as to whether *Trichuris* infecting humans (recognized as *T. trichiura*) is a distinct species from that found in pigs (recognized as *T. suis*), or not. In the present study, we sequenced, annotated and compared the complete mitochondrial genomes of *Trichuris* from these two hosts and undertook a phylogenetic analysis of the mitochondrial datasets. This analysis showed clear genetic distinctiveness and strong statistical support for the hypothesis that *T. trichiura* and *T. suis* are separate species, consistent with previous studies using nuclear ribosomal DNA sequence data. Future studies could explore, using mitochondrial genetic markers defined in the present study, cross-transmission of *Trichuris* between pigs and humans in endemic regions, and the population genetics of *T. trichiura* and *T. suis*.

## Introduction

Soil-transmitted helminths ( = geohelminths), including whipworm, are responsible for neglected tropical diseases (NTDs) of humans in developing countries [1]–[3]. *Trichuris trichiura* infects ∼600 million people worldwide. This parasite is transmitted directly via a direct, faecal-oral route. The thick-shelled (infective) eggs are ingested and then hatch, following gastric passage, in the small intestine. First-stage larvae (L1s) are released and migrate to the large intestine (caecum and colon), where they develop, following multiple moults, into adults (∼30–50 mm in length). The worms burrow their thin, thread-like anterior end into the mucosal lining of the large intestinal wall, feed on tissue fluids, mature and produce eggs. In the large intestines, large numbers of worms cause disease ( = trichuriasis), which is usually associated with entero-typhlocolitis and clinical signs, such as dysentery, bloody diarrhoea and/or rectal prolapse, in people with a high intensity of infection. Children (∼5–15 years of age) often harbour the largest numbers of worms [2]. Whipworms also infect other animal hosts, including non-human primates, pigs and dogs, and can cause clinical disease similar to trichuriasis of humans [4]–[6].

Based on current knowledge, *Trichuris* species are considered to specifically infect a particular host species or a group of related hosts. *Trichuris* species are usually identified based on host origin and the morphological features of the adult worm (spicule and pericloacal papillae) [7], [8]. However, it is not always possible to unequivocally identify and differentiate *Trichuris* species based on the morphology of adult worms alone. Importantly, *T. trichuria* cannot be unequivocally differentiated morphologically from *T. suis* or *Trichuris* from some other animals, such as non-human primates [7]. Over the years, there has been considerable discussion as to whether *T. trichuira* and *T. suis* are the same or distinct species [9]–[13], and whether humans can become infected with *T. suis*, and pigs with *T. trichiura* in endemic countries in which both host species live in close association. Although the authors of a recent molecular study suggested that *T. suis* is a separate species from *T. trichiura* [14], only a small number of specimens from one country (Spain) was used in this study, and amplicons (from the first and second internal transcribed spacers, ITS-1 and ITS-2, of nuclear ribosomal DNA) were subjected to cloning prior to sequencing, which has significant potential to lead to artefacts [15], [16]. Therefore, the findings from this study [14] need to be interpreted with some caution at this stage. Moreover, internal transcribed spacers (ITS) of nuclear ribosomal DNA might not be suited as specific markers for enoplid nematodes, because of sequence polymorphism (heterogeneity) that occurs within species (or individuals) [14], [17].

Given this heterogeneity in nuclear rDNA, barcoding from whole mitochondrial (mt) genomes (haploid) has major advantages, particularly when concatenated protein sequences derived from all coding genes are used as markers in comparative, phylogenetic-based analyses [18]–[25]. Therefore, in the present study, we (i) characterised the mt genomes of human-derived *Trichuris* and pig-derived *Trichuris*, (ii) compared these genomes and (iii) then tested the hypothesis that human-*Trichuris* and pig-*Trichuris* are genetically distinct in a phylogenetic analysis of sequence data sets representing both genomes and those from selected nematodes for comparative purposes.

## Materials and Methods

### Ethics statement

This study was approved by the Animal Ethics Committee of the Lanzhou Veterinary Research Institute, Chinese Academy of Agricultural Sciences. For the collection of *Trichuris* from humans, the subjects provided informed, written consent. All pigs, from which *Trichuris* specimens were collected, were handled in accordance with good animal practices required by the Animal Ethics Procedures and Guidelines of the People's Republic of China.

### Parasites and isolation of total genomic DNA

Adult specimens of *Trichuris* were collected from the caecum of a human patient during surgery in Zhanjiang People's Hospital in Zhanjiang, Guangdong Province, China. Adult specimens of *Trichuris* were also collected from the caecum from a pig slaughtered in an abattoir in Zhanjiang in the same province. Adult worms from each host were washed separately in physiological saline, identified morphologically [9], [26], fixed in 70% (v/v) ethanol and stored at −20°C until use. Total genomic DNA was isolated separately from two individual worms (coded Ttr2 and TsCS1 for human-*Trichuris* and pig-*Trichuris*, respectively) using an established method [27]. The region spanning ITS-1, the 5.8S gene and ITS-2 was amplified from each of these individuals by PCR using previously reported primers [14] and sequenced directly. The ITS-1 sequence of the human-*Trichuris* sample had 99.3% similarity with that of *T. trichiura* from human in Thailand (GenBank accession no. GQ352554). The ITS-1 and ITS-2 sequences of the pig-*Trichuris* sample had 98.6% and 98.5% similarity with that of *T. suis* from pigs in Spain (GenBank accession nos. AJ781762 and AJ249966, respectively) [14].

### Long-range PCR-based sequencing of mt DNA

To obtain some mt gene sequence data for primer design, we amplified regions (400–500 bp) of the *cox*1 and *nad*1 genes by using (relatively) conserved primers JB3/JB4.5 and JB11/JB12, respectively [28], and of *nad*4 and *rrn*L genes using primers designed in this study (Table 1) by PCR. The amplicons were sequenced from both directions, using BigDye terminator v3.1, ABI PRISM 3730. We then designed primers (see Table 2) to regions within *cox*1, *nad*1, *nad*4 and *rrn*L and amplified from total genomic DNA (from an individual worm) the entire mt genome in three (for human-*Trichuris*) or four (for pig-*Trichuris*) overlapping fragments (of ∼2–4 kb each) between *nad*1 and *nad*4, *nad*4 and *rrn*L, and *rrn*L and *cox*1, *cox*1 and *nad*1. The cycling conditions used were 92°C for 2 min (initial denaturation), then 92°C for 10 s (denaturation), 50°C for 30 s (annealing), and 60–68°C for 10 min (extension) for 10 cycles, followed by 92°C for 10 s, 50°C for 30 s, and 60–68°C for 10 min for 20 cycles, with a cycle elongation of 10 s for each cycle and a final extension at 60–68°C for 7 min. Each amplicon, which represented a single band in a 0.8% (w/v) agarose gel, following electrophoresis and ethidium-bromide staining, was column-purified and then sequenced using a primer walking strategy [29].

**Table 1:** Sequences of primers for amplifying short-PCR fragments from human-*Trichuris* and pig-*Trichuris*.

Primer	Sequence (5′ to 3′)	Reference
Human-*Trichuris*		
TTnad1F	CTTATATAGGTATTTCGTCAACGACG	This study
TTnad1R	TATTCATGCCTATAAATAGAAAAGCA	This study
TTnad4F	TAAGGCTCATGTWGAAGCTCCYG	This study
TTnad4R	ACCAAWGCAACAGAMGGAGGWGWAC	This study
TTrrnLF	TAAATGGCCGTCGTAACGTGACTGT	This study
TTrrnLR	AAAGAGAATCCATTCTATCTCGCAACG	This study
Pig-*Trichuris*		
JB3	TTTTTTGGGCATCCTGAGGTTTAT	[28]
JB4.5	TAAAGAAAGAACATAATGAAAATG	[28]
JB11	AGATTCGTAAGGGGCCTAATA	[28]
JB12	ACCACTAACTAATTCACTTTC	[28]
TSnad4F	TAAGGCTCATGTWGAAGCTCCYG	This study
TSnad4R	ACCAAWGCAACAGAMGGAGGWGWAC	This study
TSrrnLF	TAAATGGCCGTCGTAACGTGACTGT	This study
TSrrnLR	AAAGAGAATCCATTCTATCTCGCAACG	This study

**Table 2:** Sequences of primers for amplifying mitochondrial DNA regions from human-*Trichuris* and pig-*Trichuris*.

Primer	Sequence (5′ to 3′)	Amplified region (cf. Fig. 1)
Human-*Trichuris*		
TTnad1FTTnad4R	GGACGGACTCCATTTGATTTACT CTTCCTAGTGGGCATATTGTCTT	Partial *nad*1-(NCR-L)-K-*nad*2-M-F-*nad*5--H-R-partial *nad*4
TTnad4FTTrrnLR	GCGTAAGACAATATGCCCACTAG AAGACAATATGCCCACTAGGAAG	partial *nad*4-*nad*4L-T-P-*nad*6-*cyt*b-S_1_--*rrn*S-V-partial *rrn*L
TTrrnLFTTnad1R	CCCCAGGGATAACAGCACAATAA GTAATCTTCCAAATAAGGCGAACT	Partial *rrn*L-*atp*6-*cox*3-W-Q-I-G-D-*atp*8--*nad*3-(NCR-S)-S_2_-N-L_1_-A-C-Y-*cox*1-*cox*2-L_2_-E-partial *nad*1
Pig-*Trichuris*		
TSnad1FTSnad4R	AGCATACAGAAGAGTAACACACATA AATAACCGAGACAAACTACTAAATG	Partial *nad*1-(NCR-L)-K-nad2-M-F-*nad*5--H-R-partial *nad*4
TSnad4FTSrrnLR	ATGGTTATGTGTGTTACTCTTCTGT ATGTGTGTGTTATTCTGTACGCTGC	Partial *nad*4-*nad*4L-T-P-*nad*6-*cyt*b-S_1_--*rrn*S-V-Partial *rrn*L
TSrrnLFTScox1R	CGTCGTAACGTGACTGTGCTAAGGT TAAAGCAAATTGGGTTTTTTGAAGA	Partial *rrn*L-*atp*6-*cox*3-W-Q-I-G-D-*atp*8--*nad*3-(NCR-S)-S_2_-N-L_1_-A-C-Y-partial *cox*1
TScox1FTSnad1R	TTTTAGGTTGTTTTGTATGAGGGCA AAAAAGTGGAATCCTTCTTGTGTCA	Partial *cox*1-*cox*2- L_2_-E-partial *nad*1

### Sequence analyses

Sequences were assembled manually and aligned against the complete mt genome sequences of other nematodes (available publicly) using the computer program Clustal X 1.83 [30] to infer gene boundaries. The open-reading frames (ORFs) and codon usages of protein-coding genes were predicted using the program MacVector v.4.1.4 (Kodak), and subsequently compared with that of *Trichinella spiralis* [31]. Translation initiation and translation termination codons were identified based on comparison with those reported previously [31]. Codon usages were examined based on the relationships between the nucleotide composition of codon families and amino acid occurrence, for which codons are partitioned into AT rich codons, GC-rich codons and unbiased codons. The secondary structures of 22 tRNA genes were predicted using tRNAscan-SE [32] and/or manual adjustment [33].

### Phylogenetic analyses

Amino acid sequences inferred from the 12 protein-coding genes (i.e. not *atp*-8) common among all of the nematodes included here were concatenated into a single alignment, and then aligned with those of 9 other enoplid nematodes (*Trichinella spiralis*, GenBank accession number NC_002681; *Xiphinema americanum*, NC_005928; *Hexamermis agrotis*, NC_008828; *Agamermis* sp., NC_008231; *Romanomermis culicivorax*, NC_008640; *Romanomermis iyengari*, NC_008693; *Romanomermis nielseni*, NC_008692; *Strelkovimermis spiculatus*, NC_008047; *Thaumamermis cosgrovei*, NC_008046), using the chromadorean nematode, *Brugia malayi* (NC_004298) as the outgroup. Any regions of ambiguous alignment were excluded using Gblocks (http://molevol.cmima.csic.es/castresana/Gblocks_server.html; Talavera and Castresana 2007) using stringent selection criteria (do not allow many contiguous nonconserved positions). Phylogenetic analyses were conducted using three methods: Bayesian inference (BI) analysis was conducted with four independent Markov chain runs for 1,000,000 metropolis-coupled MCMC generations, sampling a tree every 100th generation in MrBayes 3.1.1 [34]; the first 2,500 trees represented burn-in, and the remaining trees were used to calculate Bayesian posterior probabilities (pp). Maximum likelihood (ML) analyses were performed using PhyML 3.0 [35], and the (mtREV for amino acid sequences and GTR for *rrn*L nucleotide sequences) models were determined based on the Akaike information criterion (AIC). Bootstrap support was calculated using 100 bootstrap replicates. Maximum parsimony (MP) analysis was conducted using PAUP 4.0 Beta 10 [36], with indels treated as missing character states; 1,000 random additional searches were performed using TBR. Bootstrap support was calculated using 1,000 bootstrap replicates, and 100 random taxon additions in PAUP. Phylograms were drawn using the program Tree View v.1.65 [37].

### Sequencing of *rrn*L and analysis

Two primers, *rrn*LF (5′-TAAATGGCCGTCGTAACGTGACTGT-3′) and *rrn*LR (5′- AAAGAGAATCCATTCTATCTCGCAACG-3′), were employed for PCR amplification and subsequent sequencing of a portion (471 bp for human-*Trichuris* and 482 bp for pig-*Trichuris*) of the large subunit of mt ribosomal RNA (*rrn*L) from multiple individuals of human- and pig-derived *Trichuris* (Table 3). The *rrn*L sequence from *T. spiralis* (accession number NC_002681) [31] was used as the outgroup in phylogenetic analyses, because this morphologically distinct species is related to *Trichuris* [38]. All *rrn*L sequences were aligned using Clustal X, and the alignment was modified manually, based on the predicted secondary structure of the *rrn*L for *Trichuris* [31], and then subjected to phylogenetic analysis using the same methods as described above.

**Table 3:** *Trichuris* samples from humans and pigs in different geographical locations in China.

Species	Sample codes	No. of individuals	Host	Geographical origin	Accession No.*
Human-*Trichuris*	TTGZ2-3,TTGZ5-8	6	Human	Zhanjiang, Guangdong	AM993017-AM993022
Pig-*Trichuris*	TSCS1-2	2	Pig	Changsha, Hunan	AM993023-AM993024
Pig-*Trichuris*	TSML	1	Pig	Miluo, Hunan	AM993025
Pig-*Trichuris*	TSPJ	1	Pig	Pingjiang, Hunan	AM993026
Pig-*Trichuris*	TSYY	1	Pig	Yiyang, Hunan	AM993027
Pig-*Trichuris*	TSYJ1-2	2	Pig	Yangjiang, Guangdong	AM993028-AM993029
Pig-*Trichuris*	TSZJ1-3	3	Pig	Zhanjiang, Guangdong	AM993030–AM993032

*: GenBank accession numbers of mitochondrial *rrn*L sequences of these *Trichuris* samples.

## Results

### Features of the mt genomes of *Trichuris* from the human or pig host

**Figure 1:**
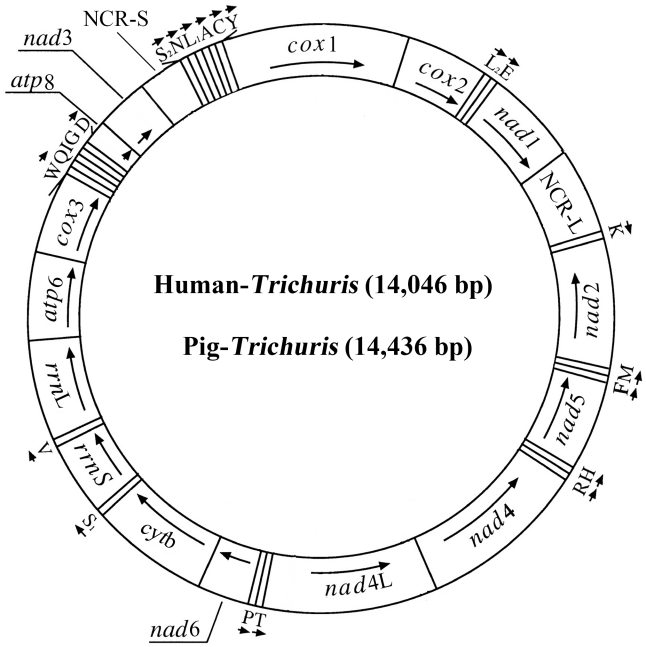
Structure of the mitochondrial genome for human-*Trichuris* and pig-*Trichuris*. Genes follow standard nomenclature [18], except for the 22 tRNA genes, which are designated using one-letter amino acid codes, with numerals differentiating each of the two leucine- and serine-specifying tRNAs (L1 and L2 for codon families CUN and UUR, respectively; S1 and S2 for codon families AGN and UCN, respectively). “NCR-L” refers to a large non-coding region; “NCR-S” refers to a small non-coding region.

The complete mt genome sequences were 14,046 nt (human-*Trichuris*) and 14,436 nt (pig-*Trichuris*) in length, respectively (GenBank accession numbers GU385218 and GU070737). Each mt genome contains 13 protein-coding genes (*cox*1-3, *nad*1-6, *nad*4L, *cyt*b, *atp*6 and *atp*8), 22 transfer RNA genes and two ribosomal RNA genes (*rrn*S and *rrn*L) (Table 4). The genes *nad*6 and *cox*3 overlapped by 25 bp (human-*Trichuris*); *nad*5 overlaps by 7 and 1 bp with tRNA-His, tRNA-Ser^(AGN)^ by 8 and 3 bp with *rrn*S, tRNA-Asp by 13 and 4 bp with *atp*8 for human-*Trichuris* and pig-*Trichuris*, respectively. The *atp*8 gene is encoded (Fig. 1), as is typical for adenophorean nematodes [31]. The protein-coding genes are transcribed in different directions, as described for *T. spiralis* and *X. americanum* [31], [39]. Except for four protein-coding genes (*nad*2, *nad*5, *nad*4 and *nad*4L) and six tRNA genes (tRNA-Arg, tRNA-His, tRNA-Met, tRNA-Phe, tRNA-Pro and tRNA-Thr) encoded on the L-strand, all other genes were encoded on the H-strand. The AT-rich regions are located between *nad*1 and tRNA-Lys, and *nad*3 and tRNA-Ser^(UCN)^, which differs from those of secernentean nematodes [19], [33]. The nucleotide composition of the entire mt genome is biased toward A and T, with T being the most favoured nucleotide and G being least favoured, which is consistent with mt genomes of some other nematodes for which mt genomic data are available [18], [19], [21], [31], [33]. The overall A+T content is 68.1% for human-*Trichuris* (33.6% A, 34.5% T; 15.0% G and 16.9% C) and 71.5% for pig-*Trichuris* (35.6% A, 35.9% T; 13.4% G and 15.1% C).

**Table 4:** Positions and nucleotide sequence lengths of mitochondrial genomes of human-*Trichuris* and pig-*Trichuris*.

Genes	Positions and nt sequence lengths (bp)		Strand	Ini/Ter codons*		Anticodons*
	Tt	Ts		Tt	Ts	Tt/Ts
*cox*1	1-1545	1-1542	H	ATG/TAA	ATG/TAG	
*cox*2	1560–2234	1578–2258	H	ATG/TAA	ATG/TAA	
tRNA-LeuUUR (L_2_)	2251-313 (63)	2271-2332 (62)	H			TAA
tRNA-Glu (E)	2318-2374 (57)	2337-2393 (57)	H			TTC
*nad*1	2397-3296	2415-3314	H	ATA/TAA	ATT/TAG	
Non-coding region (NCR-L)	3297-3458	3315-3458	H			
tRNA-Lys (K)	3459-3524 (66)	3459-3521 (63)	H			TTT
*nad*2	4406-3522	4414-3533	L	ATA/TAA	ATA/TAG	
tRNA-Met (M)	4479-4419 (61)	4485-4424 (62)	L			CAT
tRNA-Phe (F)	4530-4474 (57)	4546-4488 (59)	L			GAA
*nad*5	6078-4531	6094-4538	L	ATA/TAA	ATA/TAG	
tRNA-His (H)	6128-6072 (57)	6150-6094 (57)	L			GTG
tRNA-Arg (R)	6194-6130 (65)	6218-6152 (67)	L			ACG
*nad*4	7406-6195	7432-6224	L	ATG/TAA	ATA/TAA	
*nad*4L	7682-7425	7901-7650	L	ATA/TAA	ATA/TAG	
tRNA-Thr (T)	7744-7687 (58)	7962-7905 (58)	L			TGT
tRNA-Pro (P)	7802-7744 (59)	8023-7966 (58)	L			TGG
*nad*6	7795-8271	8016-8486	H	ATT/TAA	ATT/TAA	
*cyt*b	8278-9384	8501-9613	H	ATG/TAG	ATG/TAG	
tRNA-SerAGN (S1)	9383-9432 (50)	9612-9666 (55)	H			GCT
*rrn*S	9425-10122	9664-10375	H			
tRNA-Val (V)	10124-10180 (57)	10375-10431 (57)	H			TAC
*rrn*L	10180-11190	10440-11450	H			
*atp*6	11173-12000	11422-12249	H	ATA/TAA	ATA/TAA	
*cox*3	11975-12748	12259-13035	H	ATG/TAA	ATG/TAA	
tRNA-Trp (W)	12755-12817 (63)	13040-13106 (67)	H			TCA
tRNA-Gln (Q)	12821-12874 (54)	13110-13166 (57)	H			TTG
tRNA-Ile (I)	12871-12937 (66)	13169-13234 (66)	H			GAT
tRNA-Gly (G)	12947-13003 (57)	13253-13308 (56)	H			TCC
tRNA-Asp (D)	13009-13067 (58)	13302-13363 (62)	H			GTC
*atp*8	13055-13219	13360-13530	H	ATA/TAG	TTG/TAA	
*nad*3	13229-13570	13555-13896	H	ATA/TAA	ATA/TAA	
Non-coding region (NCR-S)	13571-13663	13887-14003	H			
tRNA-Ser UCN (S2)	13664-13715 (52)	14004-14055 (52)	H			TGA
tRNA-Asn (N)	13715-13768 (54)	14055-14113 (59)	H			GTT
tRNA-LeuCUN (L1)	13776-13842 (67)	14131-14193 (63)	H			TAG
tRNA-Ala (A)	13845-13899 (57)	14196-14250 (55)	H			TGC
tRN A-Cys (C)	13925-13979 (55)	14274-14328 (55)	H			GCA
tRNA-Tyr (Y)	13986-14046 (50)	14336-14394 (59)	H			TGT

*: Initiation and termination codons for protein-coding genes as well as their tRNA gene anticodons (starting from tRNA-T).

Tt: human-*Trichuris*; Ts: pig-*Trichuris*; Ini/Ter codons: initiation and termination codons.

### Annotation

Protein-coding genes were annotated by aligning sequences and identifying translation initiation and termination codons by comparison with inference sequences for other nematodes. For both human-*Trichuris* and pig-*Trichuris*, the lengths of protein-coding genes were in the following order: *nad*5 (1548–1557 bp) >*cox*1>*nad*4>*cyt*b>*nad*1>*nad*2>*atp*6>*cox*3>*cox*2>*nad*6>*nad*3>*nad*4L>*atp*8 (165–171 bp) (Table 4). The longest gene is *nad*5, and the lengths of the *nad*1 and *nad*3 genes are the same for human-*Trichuris* and pig-*Trichuris* (Table 5). The inferred nucleotide and amino acid sequences of each of the 13 mt proteins of human-*Trichuris* were compared with pig-*Trichuris*. For individual genes, the nucleotide and amino acid sequence differences between human-*Trichuris* and pig-*Trichuris* vary from 25.4 to 37.4% and 13.6 to 62.5%, respectively (Table 5).

**Table 5:** Differences in mitochondrial nucleotide and predicted amino acid sequences between human-*Trichuris* and pig-*Trichuris.*

Gene/region	Nucleotide sequence length		Nucleotide difference (%)	Number of aa		aa difference (%)
	Tt	Ts	Tt/Ts	Tt	Ts	Tt/Ts
*atp*6	828	828	39.6	275	275	49.5
*nad*1	900	900	33.1	299	299	33.1
*nad*2	885	882	34.1	294	293	40.8
*nad*3	342	342	33.6	113	113	31.9
*nad*4	1212	1209	40.9	403	402	58.3
*nad*4L	258	252	38.0	85	83	42.4
*nad*5	1548	1557	35.9	515	518	42.5
*nad*6	477	471	33.1	158	156	38.6
*cox*1	1545	1542	25.4	514	513	13.6
*cox*2	675	681	30.7	224	226	28.8
*cox*3	774	777	35.5	257	258	34.5
*cyt*b	1107	1113	27.7	368	370	26.2
*atp*8	165	171	47.4	54	56	62.5
All 22 *trns*	1283	1306	25.4	-	-	-
*rrn*S	698	712	24.6	-	-	-
*rrn*L	1011	1011	25.1	-	-	-
Non-coding	255	261	36.6	-	-	-

Tt: human-*Trichuris*, Ts: pig-Trichuris, Trs: *Trichinella spiralis*, aa: amino acids.

A total of 3559 and 3562 amino acids are encoded in the mt genome of human-*Trichuris* and pig-*Trichuris*, respectively. As the mt genomes of nematodes can contain non-standard initiation codons [18], the identification of initiation codons can sometimes be challenging. For human-*Trichuris*, five genes (*cox*1, *cox*2, *cox*3, *cyt*b and *nad*4) start with ATG and eight genes (*nad*1, *nad*2, *nad*3, *nad*5, *nad*6, *nad*4L, *atp*6 and *atp*8) use ATA. All genes have complete termination codons, 11 genes (*cox*1, *cox*2, *cox*3, *nad*1, *nad*2, *nad*3, *nad*4, *nad*4L, *nad*5, *nad*6 and *atp*6) use TAA and two genes (*atp*8 and *cyt*b) use TAG as a termination codon, respectively. For pig-*Trichuris*, six genes (*nad*2, *nad*3, *nad*4 *nad*4L *nad*5 and *atp*6) start with ATA, four genes (*cox*1, *cox*2, *cox*3 and *cyt*b) with ATG, and one gene (*atp*8) with TTG. All protein-coding genes have complete termination codon; seven genes (*cox*2, *cox*3, *nad*3, *nad*4, *nad*6, *atp*6 and *atp*8) stop with TAA and six genes (*cox*1, *nad*1, *nad*2, *nad*5, *nad*4L and *cyt*b) use TAG. No abbreviated stop codons, such as TA or T, were detected. Such codons are known to occur in the mt genomes of other nematodes, such as *Strongyloides stercoralis* (*cyt*b, *nad*4, *nad*1 and *atp*6) and *T. spiralis* (*cyt*b and *nad*4) and *Caenorhabditis elegans* (*nad*1 and *nad*3) [19], [31], [40]. The *atp*8 gene was inferred by comparison with the homologous gene of *T. spiralis*. The ORF of this gene was located between genes tRNA-Asp and *nad*3 and inferred by the presence of a methionine for human-*Trichuris* and a leucine for pig-*Trichuris*.

Twenty-two tRNA genes were predicted from the mt genomes of human-*Trichuris* and pig-*Trichuris* and varied from 50 to 67 nt in length. Most of the tRNA genes are smaller than the corresponding genes in the mt genomes of other nematodes due to a reduced TΨC stem-loop region (TV-replacement loop) or DHU stem-loop region [39]. Most of the tRNA gene sequences can be folded into conventional secondary four-arm cloverleaf structures. In these tRNA, there is a strict conservation of the sizes of the amino acid acceptor stem (11–15 bp) and the anticodon loop (7 bp). Their D-loops consist of 5–9 bp. The two tRNA-Ser each contain the TΨC arm and loop, but lack the DHU arm and loop.

The two ribosomal RNA genes (*rrn*L and *rrn*S) of human-*Trichuris* and pig-*Trichuris* were inferred based on comparisons with sequences from *T. spiralis*; *rrn*L is located between tRNA-Val and *atp*6, and *rrn*S is located between tRNA-Ser^(AGN)^ and tRNA-Val. The length of *rrn*L is 1011 bp for both human-*Trichuris* and pig-*Trichuris*. The lengths of the *rrn*S genes are 698 bp for human-*Trichuris* and 712 bp for pig-*Trichuris*. The A+T contents of *rrn*L for human-*Trichuris* and pig-*Trichuris* are 72.5% and 76.4%, respectively. The A+T contents of *rrn*S for human-*Trichuris* and pig-*Trichuris* are 69.9% and 75.4%, respectively.

Two AT-rich non-coding regions (NCRs) were inferred in the mt genomes of both human-*Trichuris* and pig-*Trichuris*. For these genomes, the long NCR (designated NCR-L; 162 bp and 144 bp in length, respectively) is located between the *nad*1 and tRNA-Lys (Fig. 1), has an A+T content of 71–72%. This overall A+T content is lower than those reported for nematodes (77.9–93.1%) studied to date [18], [19], [21], [33]. In this NCR, there are also 26 nt (human-*Trichuris*) and 17 nt (pig-*Trichuris*) AT dinucleotide repeats. Similar repeats have been detected in this region in *C. elegans* and *A. suum* [40]. For both human-*Trichuris* and pig-*Trichuris*, the short NCR (NCR-S; 93 bp and 117 bp in length) is located between genes *nad*3 and tRNA-Ser ^(UCN)^ (Fig. 1), with an A+T content of 65.6% and 84.6%, respectively. This region contains dinucleotide [AT]_26_ repeats and might form a hairpin loop structure (cf. AAAAAAAATTTTTTTTTT). Although nothing is yet known about the replication process in the mt DNA of parasitic nematodes, the high A+T content and the predicted structure of the AT-rich NCRs suggest an involvement in the initiation of replication [41].

#### Comparative analyses between human-*Trichuris* and pig-*Trichuris*

The full mt genome sequence of human-*Trichuris* (accession no. GU385218) was 14046 bp in length, 390 bp shorter than that of pig-*Trichuris* (accession no. GU070737). The arrangement of the mt genes (i.e., 13 protein genes, 2 *rrn* genes and 22 tRNA genes) and NCRs were the same. A comparison of the nucleotide sequences of each mt gene and NCR, as well as the amino acid sequences, conceptually translated from all protein genes of the two *Trichuris*, is given in Tables 4 and 5. The sequence lengths of individual genes and NCRs were the same for human-*Trichuris* and pig-*Trichuris*, except for variation of three to nine nucleotides in the each of 13 coding gene, 18 nucleotides in the first non-coding region and of one to nine nucleotides in each of 22 tRNA genes (Table 4). The magnitude of sequence variation in each gene and NCR between human-*Trichuris* and pig-*Trichuris* ranged from 24.6–47.4%. Sequence difference across the entire mt genome was 32.87%. The greatest variation was in the *atp*8 gene (47.4%), whereas least differences (24.6% and 25.1%) were detected in the *rrn*S and *rrn*L subunits, respectively (Table 5).

**Figure 2:**
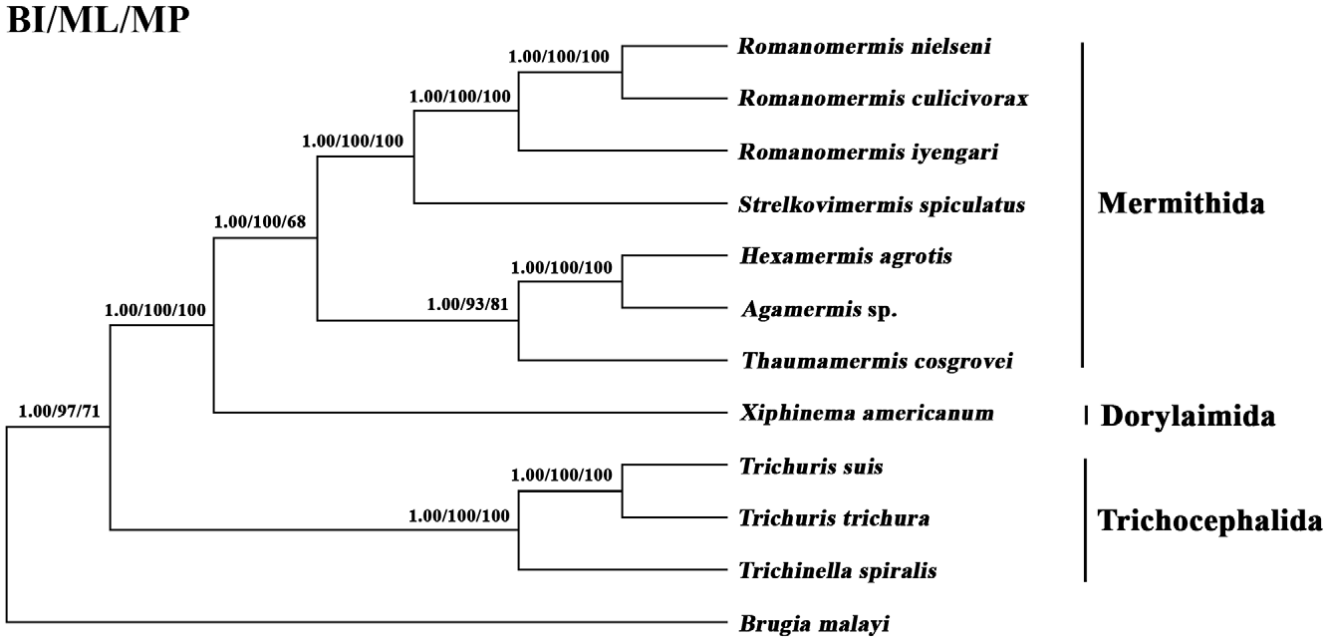
Inferred phylogenetic relationship among enoplid nematodes. The phylogenetic relationship was inferred based on the concatenated amino acid sequences of 12 protein-coding genes (with the exception of *atp*8) by Bayesian (BI), maximum likelihood (ML) and maximum parsimony (MP) analyses, using *Brugia malayi* (a chromadorean nematode) (NC_004298) as the outgroup. The numbers along branches indicate bootstrap values resulting from different analyses in the order: BI/ML/MP.

Amino acid sequences inferred from individual mt protein genes of human-*Trichuris* were compared with those of pig-*Trichuris*. The amino acid sequence differences ranged from 13.6–62.5%, with COX1 being the most conserved protein, and ATP8 the least conserved. There were 1299 amino acid substitutions (for an alignment length of 3559 and 3562 positions) in the 13 proteins, the majority of which were in the proteins NAD4 (n = 235), NAD5 (n = 220) and NAD2 (n = 120). Of the 1299 amino acid substitutions, 407 (31.3%) represented potentially informative characters, the greatest number of them being in the proteins NAD1 (n = 84), NAD5 (n = 55) and ATP6 (n = 37). The phylogenetic analyses of amino acid sequence datasets using *B. malayi* as the outgroup reflected the clear genetic distinctiveness between human-*Trichuris* and pig-*Trichuris* and also the grouping of these two members of *Trichuris* with *T. spiralis* (Trichocephalida), with absolute support, to the exclusion of members of the Dorylaimida and Mermithida (Fig. 2).

Comparison of the mt genomes of human-*Trichuris* and pig-*Trichuris* showed that the *rrn*S and *rrn*L were the two most conserved genes (Table 5). Sequence variation in part of the *rrn*L gene was assessed among 16 individuals of *Trichuris* from humans and pigs (Table 3). Sequences of the 6 human-*Trichuris* individuals were of the same length (419 bp). Nucleotide variation among the 6 human-*Trichuris* individuals was detected at 4 sites (sequence positions 206, 228, 233 and 274; GenBank accession numbers AM993017–AM993022). Sequences of the 10 pig-*Trichuris* individuals were of the same length (430 bp). Nucleotide variation also occurred at 4 sites (sequence positions 184, 233, 318 and 395, GenBank accession numbers AM993023–AM993032).

**Figure 3:**
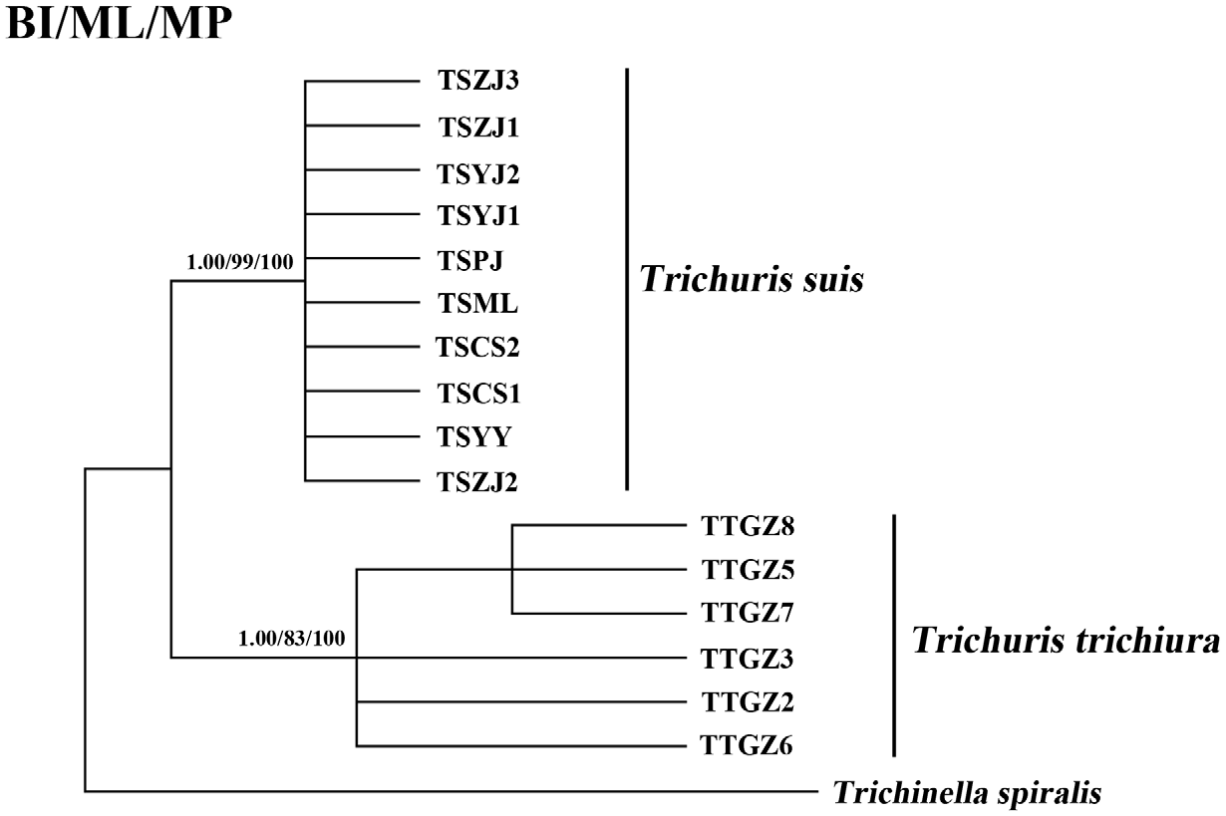
Inferred phylogenetic relationship among representative *Trichuris* samples from humans and pigs. The phylogenetic relationship was inferred by Bayesian (BI), maximum likelihood (ML) and maximum parsimony (MP) analyses of mitochondrial *rrn*L sequence data, using *Trichinella spirialis* as the outgroup. The numbers along branches indicate bootstrap values from different analyses in the order: BI/ML/MP.

The alignment of the partial *rrn*L sequences revealed that all individuals of human-*Trichuris* differed at 89 nucleotide positions when compared with pig-*Trichuris*. These differences included 15 indels, 16 purine transitions (A<->G) and 19 transversion (A<->T). Phylogenetic analyses of the *rrn*L sequence data revealed strong support for the separation of human-*Trichuris* from pig-*Trichuris* individuals into two distinct clades, and the trees produced using the three different methods were essentially the same in topology (Fig. 3). Sixteen of the 89 nucleotide differences were considered as derived (i.e., autapomorphic) characters, using *T. spiralis* as the outgroup.

## Discussion

A substantial level of nucleotide difference (32.9%) was detected in the complete mt genome between an individual of human-*Trichuris* and pig-*Trichuris* from China. The sequence variation detected in the 13 protein-coding genes (25.4–47.4%) and in NCRs (36.6%) was consistent with previous findings of variation in the nucleotide sequences of the nuclear ITS rDNA from human and pig [14]. However, for many nematodes [42], [43], there is usually greater within-species variation in mt protein-coding genes than in the ITS. For example, the magnitude of the nucleotide sequence variation in the 12 common mt protein genes (3–7%) [20] was greater than the 15 (1.8%) variable positions in the ITS (over 852 bp) detected among multiple individuals of the human hookworm, *N. americanus* [44].

Comparison between human- and pig-derived *Trichuris* from China also revealed variation at 1299 amino acid positions in the 13 predicted mt protein sequences. This level of amino acid variation (36.4%) is very high, given that mt proteins are usually conserved within a species due to structural and functional constraints [45]. In addition, previous studies of other nematodes have detected little to no within-species variation in protein sequences. For example, no within-species variation was detected in a COX1 region of 131 amino acids for *N. americanus* and for related hookworms, including *A. caninum Ancylostoma* and *A. duodenale* [24], [33]. Similarly, amino acid substitutions were recorded at only two of 196 (1%) positions (based on a comparison of conceptually translated sequences originating from GenBank accession nos. AF303135-AF303159) in partial COX1 among 151 *N. americanus* samples from four locations in China [46]. In the present study, the greatest numbers of amino acid differences between human-*Trichuris* and pig-*Trichuris* were in the NAD4 (n = 235; 40.9%), NAD5 (n = 220; 35.9%) and NAD2 (n = 120; 34.1%) sequences; these percentages were significantly higher than that (4.9–10%) between the two hookworms *A. caninum* and *A. duodenale* [24], [33]. The nature, extent and significance of the amino acid sequence variation between *Trichuris* from the human and pig hosts and from different geographical origins needs to be evaluated further, because there is virtually no published data on the magnitude of within-species variation in mt protein sequences for members of the genus *Trichuris*.

Genetic variation between human- and pig-*Trichuris* was also detected here in the two mt ribosomal RNA gene subunits (*rrn*L and *rrn*S). These subunits are usually more conserved in sequence than the protein genes [45], which is also supported by the present data. Comparison of the complete mt genomic data set between the two *Trichuris* individuals (Ttr2 and TsCS1) displayed less sequence variation in *rrn*S and *rrn*L (24.6% and 25.1%) compared with most protein genes (25.4–47.4%) and the non-coding regions (36.6%) (Table 5). A region (∼430 bp) in the conserved *rrn*L gene was used to examine the magnitude of genetic variation in *Trichuris* between the two different host species. A comparison of the partial *rrn*L sequences among 16 *Trichuris* individuals revealed 89 (20.7%) variable positions between human-*Trichuris* and pig-*Trichuris*, which is comparable with previous findings of a significant genetic difference (17%) in nuclear ITS between the two operational taxonomic units (OTUs) in Spain [14]. Taken together, the molecular evidence presented here supports the hypothesis that the gene pools of human-*Trichuris* and pig-*Trichuris* have been isolated for a substantial period of time and that they represent distinct species. In spite of the genetic distinctiveness recorded here between them, host affiliation is not strict [47]. Cross-infection of *Trichuris* between humans and pigs (both directions) has been described, but infection in the heterologous host is usually abbreviated [47].

In spite of the compelling evidence of genetic distinctiveness between *Trichuris* specimens from human and pig hosts, interpretation from this study needs to be somewhat guarded until detailed population genetic investigations have been conducted. Future studies could (i) explore, in detail, nucleotide variation in ribosomal and mt DNAs within and among *Trichuris* populations from humans and pigs from a range of different countries employing, for example, mutation scanning-coupled sequencing [27], (ii) establish, using accurate molecular tools, whether there is a particular affiliation between *Trichuris* and host in endemic regions and whether cross-host species infection is common or not, and (iii) attempt to establish an experimental infection of *Trichuris* of human origin in pigs, in order to be able to investigate the genetic and reproductive relationships between human-*Trichuris* and pig-*Trichuris*. Moreover, given the advent of high throughput genomic sequencing technologies, and the recent success in sequencing the nuclear genomes of the parasitic nematodes, *B. malayi* [48] and *Ascaris suum* [49], it is conceivable that the genomes of human-*Trichuris* and pig-*Trichuris* will be characterized in the near future. The transcriptome, and inferred proteome, characterised recently [50] will assist in future efforts to decode these genomes. Such work will pave the way for future fundamental molecular explorations and the design of new methods for the treatment and control of one of the world's socio-economically important nematodes [3]. This focus is important, given the impact of *Trichuris* and other soil-transmitted helminths (STHs), which affect billions of people and animals world-wide. Although *Trichuris* species are seriously neglected, genomics and related approaches provide new opportunities for the discovery of novel intervention strategies, with major implications for improving animal and human health and well being globally. In addition, the implications of genomic studies could also be highly relevant in relation to finding new treatments for immune-pathological diseases of humans [50]. Interestingly, various studies [51]–[55] have indicated that iatrogenic infections of human patients suffering from immunological disorders (such as inflammatory bowel disease, IBD) with nematodes, such as pig-*Trichuris* eggs can significantly suppress clinical symptoms. Although the mechanisms by which *Trichuris* modulates the human immune system are still unclear [52], [56], [57], studies have proposed that a modified CD4+ T helper 2 (Th2)-immune response and the production of anti-inflammatory cytokines, such the interleukins (IL-) IL-4 and IL-10, contribute to the inhibition of effector mechanisms [56], [58], [59]. Therefore, detailed investigations of pig-*Trichuris* at the molecular level could provide enormous scope for studying immuno-molecular mechanisms that take place between the parasite and humans affected by autoimmune or other immune diseases. The mt genetic markers defined in the present study should be useful to verify the specific identity of *Trichuris* employed in such studies.
